# The Non-Peptide Arginine-Vasopressin v_1a_ Selective Receptor Antagonist, SR49059, Blocks the Rewarding, Prosocial, and Anxiolytic Effects of 3,4-Methylenedioxymethamphetamine and Its Derivatives in Zebra Fish

**DOI:** 10.3389/fpsyt.2017.00146

**Published:** 2017-08-14

**Authors:** Luisa Ponzoni, Daniela Braida, Gianpietro Bondiolotti, Mariaelvina Sala

**Affiliations:** ^1^Fondazione Umberto Veronesi, Milan, Italy; ^2^Department of Medical Biotechnology and Translational Medicine (BIOMETRA), Università degli Studi di Milano, Milan, Italy; ^3^Institute of Neuroscience, Consiglio Nazionale delle Ricerche (CNR), Milan, Italy

**Keywords:** phenethylamines, zebra fish, isotocin, social preference, hallucinogens, novel tank diving test, light–dark test

## Abstract

3,4-Methylenedioxymethamphetamine (MDMA) and its derivatives, 2,5-dimethoxy-4-bromo-amphetamine hydrobromide (DOB) and *para*-methoxyamphetamine (PMA), are recreational drugs whose pharmacological effects have recently been attributed to serotonin 5HT_2A/C_ receptors. However, there is growing evidence that the oxytocin (OT)/vasopressin system can modulate some the effects of MDMA. In this study, MDMA (2.5–10 mg/kg), DOB (0.5 mg/kg), or PMA (0.005, 0.1, or 0.25 mg/kg) were administered intramuscularly to adult zebra fish, alone or in combination with the V_1a_ vasopressin antagonist, SR49059 (0.01–1 ng/kg), before carrying out conditioned place preference (CPP), social preference, novel tank diving, and light–dark tests in order to evaluate subsequent rewarding, social, and emotional-like behavior. The combination of SR49059 and each drug progressively blocked: (1) rewarding behavior as measured by CPP in terms of time spent in drug-paired compartment; (2) prosocial effects measured on the basis of the time spent in the proximity of a nacre fish picture; and (3) anxiolytic effects in terms of the time spent in the upper half of the novel tank and in the white compartment of the tank used for the light–dark test. Antagonism was obtained at SR49059 doses which, when given alone, did not change motor function. In comparison with a control group, receiving vehicle alone, there was a three to five times increase in the brain release of isotocin (the analog of OT in fish) after treatment with the most active doses of MDMA (10 mg/kg), DOB (0.5 mg/kg), and PMA (0.1 mg/kg) as evaluated by means of bioanalytical reversed-phase high-performance liquid chromatography. Taken together, these findings show that the OT/vasopressin system is involved in the rewarding, prosocial, and anxiolytic effects of MDMA, DOB, and PMA in zebra fish and underline the association between this system and the behavioral alterations associated with disorders related to substance abuse.

## Introduction

New psychoactive substances are available in various formulations and are mainly used as legal substitutes for traditional drugs of abuse. One of the largest and most important groups are psychostimulants, which affect a range of behavioral patterns in humans (1) and laboratory animal models (2, 3). It has been demonstrated that the repeated administration of psychostimulants to rodents (2, 4–8) and humans (9) can lead to addiction, induce changes in emotional states such as fear, anxiety, and depression, interfere with social behavior, and cause cognitive impairment. It has also been found that the repeated administration of cocaine and methamphetamine are anxiogenic in mice performing the elevated plus maze task (2), lead to cognitive deficit in rats when using the novel object recognition test, and induce depressive-like behavior as evaluated by the forced swimming task (4). The repeated administration of 3,4-methylenedioxymethamphetamine (MDMA) decreases social investigation and increases anxiety-like behavior (5). Rats that are prenatally exposed to (6), or neonatally treated with methamphetamine (P11-P15) (7), and subsequently given a subthreshold dose of methamphetamine in adulthood show impaired working memory. Although no working memory deficit has yet been documented in rats prenatally exposed to MDMA in the Morris water maze when a fixed platform schedule is used, they do show perseverative behavior using a cued platform schedule (8). Finally, the repeated use of MDMA by humans has been associated with sleep, mood, and anxiety disturbances, increased impulsiveness, memory deficits and attention problems, which may persist for up to 2 years after cessation (9).

Increasing attention has been given to MDMA and its phenetylamine derivatives over the last few years because, although illegal, they are sold openly through internet websites (10–15) despite their significant toxicity (13, 16, 17). It is known that MDMA is prosocial and enhances empathy in humans (18), but it evokes hyperlocomotion and anxiety in rodents (5, 19, 20) and, depending on the dose, can have an anxiogenic or anxiolytic effect on mice, rats, and zebra fish (21–23). However, there has been controversy concerning its effects on anxiety in humans as reduced anxiety has been observed in a clinical setting (24), whereas Schifano (25) found a high level of anxiety after chronic use.

Among the MDMA derivatives, 2,5-dimethoxy-4-bromo-amphetamine hydrobromide (DOB) and *para*-methoxyamphetamine (PMA) are widely used as substitutes in “ecstasy” tablets because of their similarity to MDMA (26).

2,5-Dimethoxy-4-bromo-amphetamine hydrobromide was first synthesized by Shulgin and Shulgin (27), and its recreational use increased in the mid-1980s as it was best alternative to LSD and psilocybin. Its psychoactive effects are mediated by 5HT_2A/2C_ receptor interactions within the central nervous system (28–30) and are similar to those of other hallucinogenic phenylalkylamines such as mescaline (30, 31). At an oral dose of 2 mg, it is emotionally stimulating and enhances perceptions without giving rise to perceptual distortions or hallucinations (32); however, the uncontrolled illegal use of higher doses may cause hallucinations, panic, vasospasms, coma, and even death (29–32). DOB-related fatal and non-fatal intoxication has also been reported (32–36).

*Para*-methoxyamphetamine is cheaper and more readily available than MDMA, which it was designed to replace. However, as suggested by its street name of “death,” PMA poisoning has been reported in various countries (37, 38). It is stronger than MDMA, but users tend to take more because it takes longer to act (39). In humans, it induces life-threatening hyperthermia, rhabdomyolysis, breathing difficulties, and acute renal failure (40); in rats, its acute administration increases dopamine (DA) and 5HT release in the striatum, nucleus accumbens (NAc) and frontal cortex (41). Despite their well-documented toxicity, MDMA and hallucinogens such as psylocybin, have recently been proposed as new treatments for alcohol addiction, post-traumatic stress disorder, anxiety, and depression (27, 42).

Zebra fish (*Danio rerio*) is a valuable model for high-throughput drug discovery and screening and can be used to investigate some aspects of neuropsychiatric disorders, including hallucinogen-evoked states (43). It has been found that MDMA and hallucinogens such as salvinorin A are rewarding for zebra fish undergoing the conditioned place preference (CPP) test, a widely used means of evaluating rewarding effects of different compounds (44, 45). In addition, the innate tendency of zebra fish to form shoals has often been used to examine the effects of drugs on social preference, and it has been shown that LSD, ibogaine, PCP, MK801, and MDMA markedly decrease shoal cohesion (44). It is also possible to assess anxiety by analyzing the habituation of zebra fish to novelty (46) and their response to brightly lit environments (47). The novel tank test has shown that zebra fish exposed to various doses of MDMA, LSD, mescaline, ibogaine, phencyclidine, and ketamine show anxiolytic-like responses: i.e., they spend a longer time in the upper half of the tank and there is a reduction in the latency to get to the top of the tank (48–52). The light–dark test has shown no change in zebra fish exposed to LSD (52) or psylocybin (43), but an anxiolytic effect in those exposed to ibogaine (49).

The recently discovered, dose-dependent rewarding, anxiolytic, and prosocial effects of MDMA, DOB, and PMA on zebra fish (23, 53) can be completely blocked by ritanserin, thus suggesting the involvement of 5-HT_2A/C_ receptors. However, there is growing evidence that the neuropeptide oxytocin (OT) can modulate drug-related reward and may act as a pharmacological treatment of drug dependence (54). Accordingly, use of the CPP or self-administration paradigm has shown that peripheral OT injections in mice, or intracerebroventricular (ICV) OT microinjections into mouse NAc or subthalamic nucleus, attenuate methamphetamine-induced reward (55). It is also interesting to note that the prosocial effect of MDMA has been related to central OT release in both rat and human studies (56, 57).

The characteristic changes in rat adjacent lying and anogenital sniffing induced by MDMA, vasopressin (AVP), and OT can be reversed by pretreatment with SR49059 (56), thus indicating that OT and AVP may directly act on V_1a_ vasopressin receptors to induce prosocial effects. At the same time, MDMA may indirectly stimulate V_1a_ vasopressin receptors as a result of serotonin-induced OT and/or AVP release in the hypothalamus (58).

It has emerged that nonapeptides of the vasotocin family are key regulators of social behavior in a wide range of vertebrate species: AVP and OT in mammals, and arginine vasotocin (AVT) and isotocin (IT) in teleosts (59). In order to investigate the mechanism(s) underlying the activity of amphetamine derivatives and the possibility that some of the effects of MDMA, DOB, and PMA are influenced by IT and AVT, we tested the effects of the selective V_1a_ vasopressin receptor antagonist, SR49059, on various aspects of behavior that can be successfully evaluated in zebra fish: i.e., reward, anxiety, and social behavior. We also investigated the changes in IT release within the brain after treatment with these drugs.

## Materials and Methods

### Animals

Adult wild-type short-finned zebra fish (*D. rerio*) of different genetic backgrounds (weight 0.4–1 g; age 6 and 12 months) were obtained from a local aquarium supplier (Aquarium Center, Milan, Italy). The males and females were distinguished as previously described (60, 61) and were used in a 1:1 ratio in all of the experiments. The fish were kept at a temperature of 28°C using a 14-h (from 8.00 am to 10.00 pm) light and 10-h dark cycle and were fed daily with brine shrimp and flake fish food [tropical fish food, Consorzio G5, Casatenovo (LC), Italy]. The tank water consists of deionized H2O and sea salts (0.6 g/10 L of water; Instant Ocean, Aquarium Systems, Sarrebourg, France). Each home tanks contained about 30 fish each and were constantly filtered and aerated. All of the fish were drug naive, and each fish was only used once. The experiments were started one month after the fish arrived in the laboratory in order to minimize stress, and they were habituated to the experimental apparatus for 1 h a day during the week preceding the experiments. During the experiments, the observer was blinded to the treatment allocation and sat 2 m from the tank. Behavioral testing was carried out between 9.00 am and 2.00 pm, using 10 fish per group. All of the tests were video-recorded (Canon Digital MV900) for subsequent video-aided analysis by trained observers who were also blinded to the treatments. The experiments were carried out in accordance with European Community Council Directive No. 86/609/EEC, and the subsequent Italian law governing the protection of animals used for experimental and other scientific purposes. The experimental protocol was approved in accordance with Italian Governmental Decree Nos. 18/2013 and 34/2017. Every effort was made to minimize the number of animals used and their discomfort.

### Drugs

**Figure 1:**
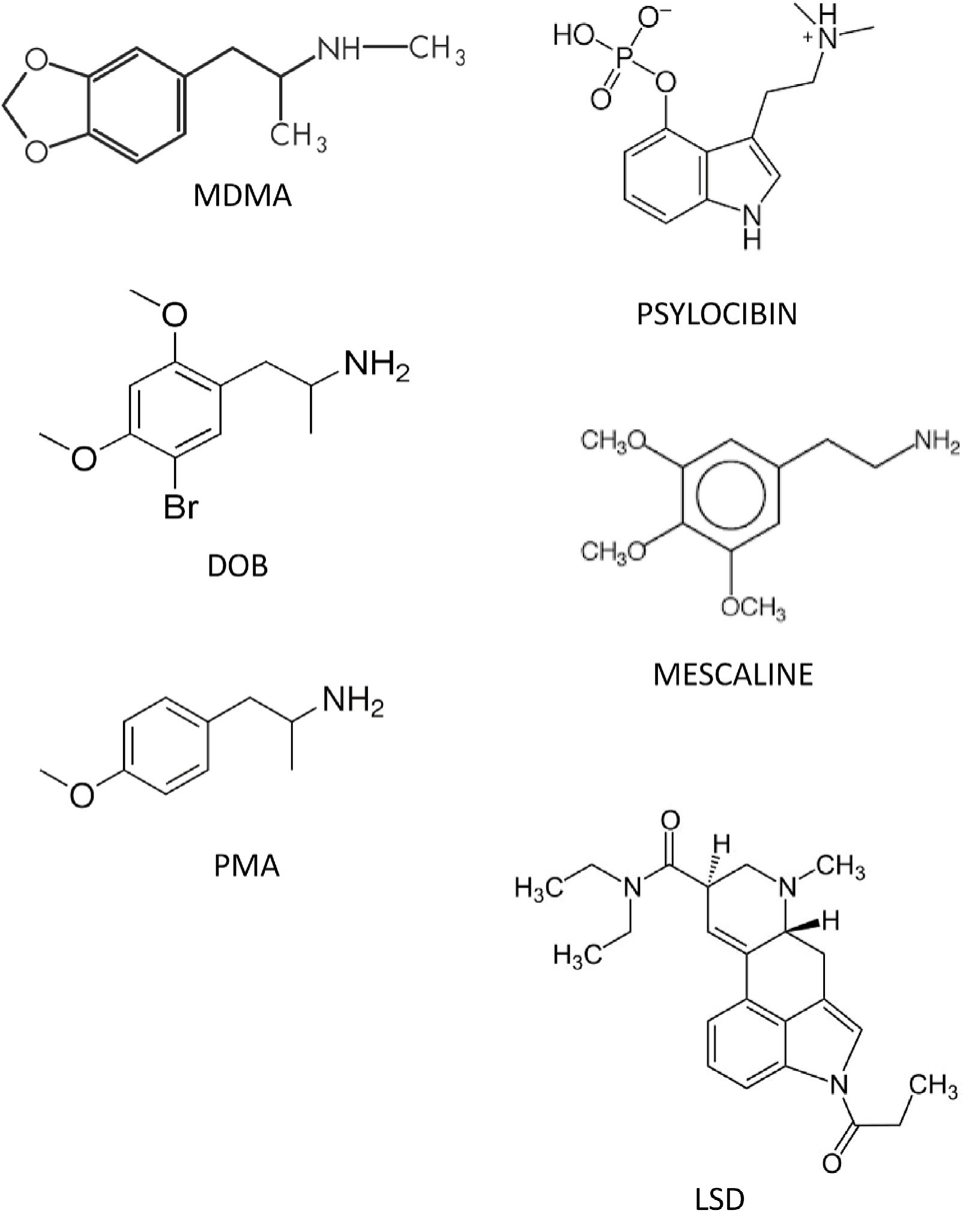
Structures of some of the phenetylamines used in the study and the main serotonergic hallucinogens.

Figure 1 shows the chemical structures of MDMA, DOB, and PMA (Sigma-Aldrich, St. Louis, MO, USA), which were dissolved in sterile saline at doses of, respectively, 10, 0.5, and 0.1 mg/kg and administered intramuscularly (IM) 5 min before each test. The doses were chosen on the basis of their known ability to maximize the drugs’ rewarding, social, and anxiolytic effects (23, 53). Given the unavailability of specific antagonists of zebra fish IT/AVT receptor subtypes and selective OT antagonists, we used (2S)-1-[[(2R,3S)5-chloro-3(2-chlorophenyl)-1-[(3,4-dimethoxyphenyl)sulfonyl]-2,3-dihydro-3hydroxy-1H-indol-2-l]carbonyl]-2-pyrrolidine carboxamide (SR49059; Sigma-Aldrich, St. Louis, MO, USA), the most selective antagonist of human and rat vasopressin V_1a_ receptors (60), at doses of 0.01, 0.1, and 1 mg/kg, which are known to block the prosocial effects of IT and AVT in zebra fish (62). When multiple treatments were required, the drug solutions were put into the same syringe in order to avoid unnecessary tissue trauma. The drug doses were calculated as salts, and all of the drugs were freshly prepared on a daily basis. The control group received saline 2 µL/g.

### Treatments

The injections were given along the posterior axis of the caudal musculature (61), in the area below the caudal fin on the left side of each fish, using a Hamilton syringe (Hamilton Bonaduz AG, Bonaduz, Switzerland).

### Conditioned Place Preference

**Figure 2:**
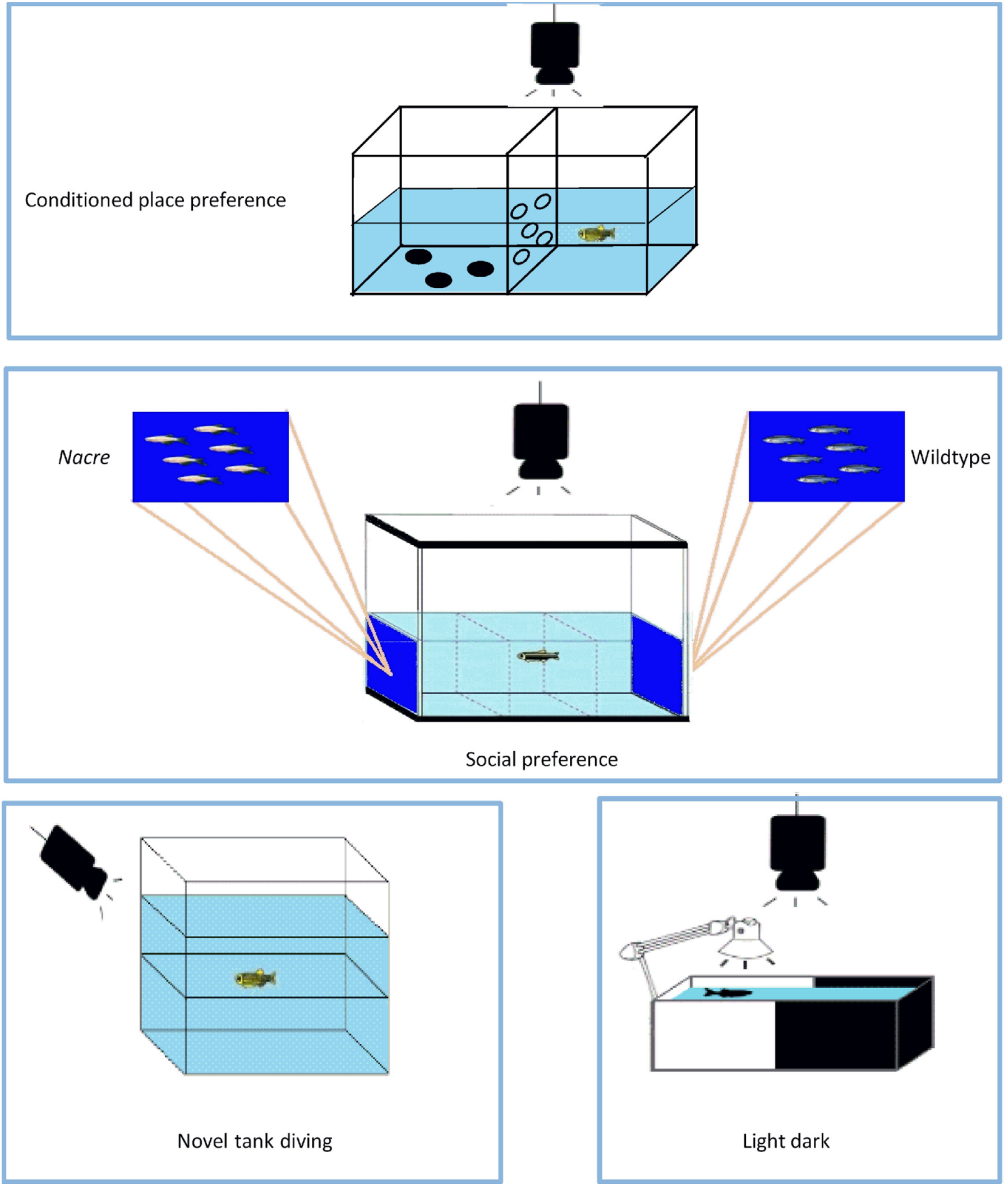
The apparatuses used in the study. The conditioned place preference test apparatus was used to evaluate the rewarding effects of 3,4-methylenedioxymethamphetamine (MDMA), 2,5-dimethoxy-4-bromo-amphetamine hydrobromide (DOB), and *para*-methoxyamphetamine (PMA). The social preference test apparatus was used to evaluate shoaling behavior. Anxiety-like behavior was studied using the novel tank diving and light–dark tests. For further explanation, see Material and Methods.

The fish were tested in a 10 cm × 20 cm × 15 cm tank divided into two 10 cm × 10 cm halves, one of which had three black polka dots on the bottom; the two halves were separated by a perforated wall that allows the fish to pass from one side to the other, albeit in a slightly obstructed manner as previously described (45) (Figure 2). During the week before the start of the CPP test, the fish were given subcutaneous injections of colored dyes (Sigma-Aldrich) so that they could be easily distinguished, as suggested by Cheung et al. (63). On the first day, after the fish had been introduced to the tank, baseline preference was established by recording the percentage of time spent in one side or the other during a 15-min trial (the preconditioning phase). After 6 h, the fish were intramuscularly injected with the different drugs, and then confined to the least preferred side for 30 min. After 24 h, the fish receiving the vehicle were confined to the opposite compartment for 30 min. The drug-texture pairings were always counterbalanced. On the third day (postconditioning phase), the fish were allowed to pass freely from one side of the tank to the other for 15 min, and the time spent in each half was recorded offline. The rewarding or aversive effects of the drugs on the fish were determined by subtracting the baseline values from the final values.

### Social Behavior

The shoaling preference test was carried out as previously described (23) in a glass tank that was 122 cm long, 55 cm high, and 32 cm wide and divided into three equal compartments (Figure 2). There was a picture of six 3 cm long nacre fish on a blue background on the left side of the tank and an unaltered picture of six zebra fish [taken from Ref. (64)] on the right side of the tank, and the lateral compartments (or stimulus areas) were separated from the central compartment. The glass tank was divided into three zones of equal volume: a left preference area, a central no-preference area, and a right preference area. The tank had two 250 W halogen lamps above either side of the tank, the light of which reflected off two sheets of Teflon positioned at an angle of 45° on the top of the tank in order to ensure that the whole of the tank received even, full-spectrum lighting. The depth of the water was 25 cm. At the beginning of the experimental trials, thin sheets of opaque plastic were placed on either side of the central compartment to act as temporary visual barriers and, immediately after undergoing drug treatment, each fish was placed in the central compartment and allowed 5 min to acclimatize. The opaque barriers were then removed and, when a fish swam parallel to one of the shoal members, it was assumed it had recognized the stimulus shoal (65). The fish were given up to 15 min to recognize both stimuli and, if they failed to do so, the test was postponed until the following week: only 0.1% of the fish failed to recognize both stimuli within the first 5 min. Shoaling preference was quantified by recording the total time each fish spent in proximity to each stimulus shoal within a period of 5 min. The data were expressed as the difference (Δ) between the time spent close to the nacre and wild-type fish pictures.

### Novel Tank Diving Test

The novel tank diving test of Egan et al. (66) was used to evaluate the anxiety-like behavior evoked by novelty. After a 1-h period of acclimatization in the experimental room, each fish was gently transferred to a transparent 1.5-L tank that had a line drawn on the outside walls midway between the surface of the water and the bottom of the tank (Figure 2). Zebra fish typically have vertical exploratory behaviors that gradually tend to increase over time (44, 66). The time spent in the upper and lower halves during the first 5 min was recorded, together with the number of times the fish moved from the lower to the upper half.

### Light–Dark Test

In order to investigate anxiety-related behaviors further, the fish underwent a previously described light/dark test (23). The test apparatus consisted of a rectangular half-black, half-white acrylic tank (20 cm × 10 cm × 15 cm) separated by a gray divider (Figure 2), and filled with water to a depth of 13 cm. The room was lit overhead (250 lux), and a 9-watt lamp was positioned above the white half of the tank in order to ensure further constant, uniform and shadow-free lighting. After being individually placed in the white compartment and allowed to acclimatize for 5 min, the divider was removed and the fish were given 5 min to swim freely between the compartments. The analyzed parameters were the time spent in the white compartment and the number of crossings between the two compartments, and the data were expressed as the difference between the time spent in each compartment. Greater exploration of the white compartment reflects a low state of anxiety.

### Brain IT Assay

The brain IT assay was restricted to the fish receiving the maximum active doses of the compounds alone, and those receiving MDMA combined with SR49059. Five minutes after being intramuscularly injected with vehicle, MDMA 10 mg/kg, PMA 0.1 mg/kg, DOB 0.5 mg/kg alone, or MDMA 10 mg/kg + SR49059 1 mg/kg, the fish were sacrificed by means of an overdose of tricaine solution (500–1,000 mg/L), and their whole brains were removed within 2 min of death. Each brain was weighed, immediately frozen on dry ice, and then stored at −80°C until analysis. IT concentrations were measured by means of bio-analytical, reversed-phase high-performance liquid chromatography with fluorescence detection. Solid-phase extraction and peptide derivatization were carried out as described in Ref. (67). The chromatographic equipment consisted of an Agilent 1100 series system (Agilent Technologies, Inc., Santa Clara, CA, USA) and Ascentis Express C_18_ column (4.6 mm × 150 mm, particle size 2.7 µm) operating in isocratic mode using acetonitrile and 0.05 M Na_2_ HPO_4⋅_H_2_O (80:20 v/v) as mobile phase (0.4 mL/min). The eluent was monitored at 530 nm (470 nm excitation).

### Statistical Analysis

The data are expressed as mean values ± the standard error of the mean (SEM). Between-group differences were assessed using one-way analysis of variance (ANOVA) for repeated measures followed by Tukey’s *post hoc* test. All of the statistical analyses were made using Prism 6 software (GraphPad Inc., La Jolla, CA, USA).

## Results

### Conditioned Place Preference

**Figure 3:**
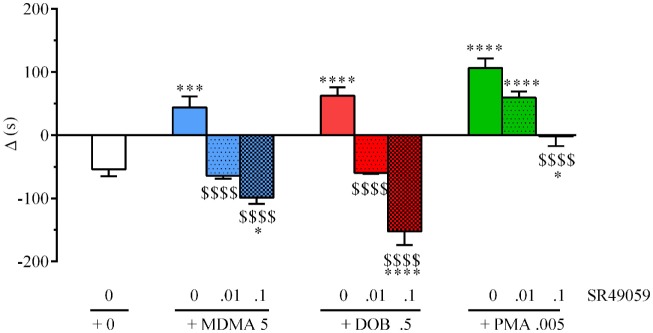
SR49050 blocks 3,4-methylenedioxymethamphetamine (MDMA)-, 2,5-dimethoxy-4-bromo-amphetamine hydrobromide (DOB)-, and *para*-methoxyamphetamine (PMA)-induced conditioned place preference (CPP). Mean values ± SEM of differences in the time spent (s) by each zebra fish in the drug-paired compartment before and after conditioning. Each drug (mg/kg) plus SR49059 (ng/kg) or vehicle was administered intramuscularly (IM) immediately before the conditioning session. *n* = 10 fish per group. *p < 0.05, **p < 0.01, ***p < 0.001 vs. the vehicle group (0 + 0); ^$^*p* < 0.05, ^$$$^*p* < 0.001 vs. the corresponding drug alone (Tukey’s *post hoc* test).

During the preconditioning phase, a simple paired *t* test revealed no significant difference between the time spent in the polka dot chamber (432.8 ± 15.3 s) and dot-free compartment (469.2 ± 14.84 s) (*t*_128_ = 0.236, *p* = 0.81). One-way ANOVA revealed between-group differences after treatment with MDMA, PMA, or DOB alone or in combination with SR49059: MDMA (*F*_3, 36_ = 4.82, *p* = 0.01), DOB (*F*_3, 36_ = 6.94, *p* = 0.003), PMA (*F*_3, 36_ = 5.57, *p* = 0.007) (Figure 3). *Post hoc* analysis showed a significant increase in postconditioning time after treatment with MDMA 10 mg/kg, DOB 0.5 mg/kg, or PMA 0.1 mg/kg, whereas SR49059 completely blocked MDMA-, DOB-, and PMA-induced CPP. SR49059 antagonism was obtained at doses that did not affect CPP, except for the highest dose that induced a rewarding effect (*F*_3, 36_ = 8.00, *p* = 0.0003) (Table 1).

**Table 1:** Effect of SR40059 on different behaviors in zebra fish.

Treatment	Dose (ng/kg)	CPP (postconditioning–preconditioning) (s)
Vehicle	0	28.48 ± 46.01
SR49059	0.01	−50.33 ± 42.06
SR49059	0.1	95.2 ± 23.83
SR49059	1	183.3 ± 0.91^*,$^

**Treatment**	**Dose (ng/kg)**	**Social preference (time)**

Vehicle	0	−54.25 ± 10.90
SR49059	0.01	−103.70 ± 4.25
SR49059	0.1	−49.50 ± 17.00

*Mean values ± SEM*.

*SR49059 was given intramuscularly 5 min before the start of each test (*n* = 10 for each dose)*.

*p < 0.05 vs. corresponding vehicle; ^$^*p* < 0.01 vs. SR49059 (one-way ANOVA, Tukey’s test).

### Social Preference

**Figure 4:**
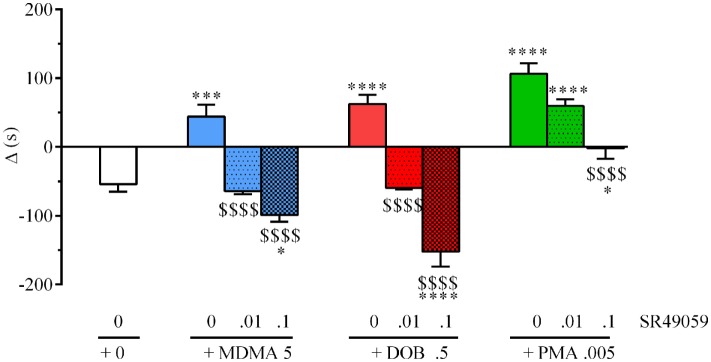
SR49050 dose-dependently antagonizes 3,4-methylenedioxymethamphetamine (MDMA)-, 2,5-dimethoxy-4-bromo-amphetamine hydrobromide (DOB)-, and *para*-methoxyamphetamine (PMA)-induced social preference in zebra fish. Mean values ± SEM of the differences in the time spent (s) in the compartment near to the nacre picture and the time spent near to the WT picture (Δ). The combination of SR49059 (ng/kg) or vehicle and each drug (mg/kg) was given intramuscularly (IM) immediately before the test. *n* = 10 fish per group. *p < 0.05, ***p < 0.001, ****p < 0.0001 vs. saline (0 + 0); ^$$$$^ *p* < 0.001 vs. the corresponding drug alone (Tukey’s test).

There were significant between-group differences in the time spent by each fish near the con-specific or nacre picture when SR49059 or vehicle were given in combination with the different drugs: MDMA (*F*_3, 36_ = 29.82, *p* < 0.0001), DOB (*F*_3, 36_ = 39.67, *p* < 0.0001), and PMA (*F*_3, 36_ = 30.58, *p* < 0.0001) (Figure 4). *Post hoc* comparisons showed that all of the drugs given alone significantly increased the time spent near the nacre picture in comparison with the vehicle group. SR49059 blocked the social preference induced by MDMA, DOB, or PMA in a dose-dependent manner. SR49059 antagonism was obtained at doses that did not affect social preference (*F*_3, 36_ = 1.14, *p* = 0.36) (Table 1).

### Novel Tank Diving Test

**Figure 5:**
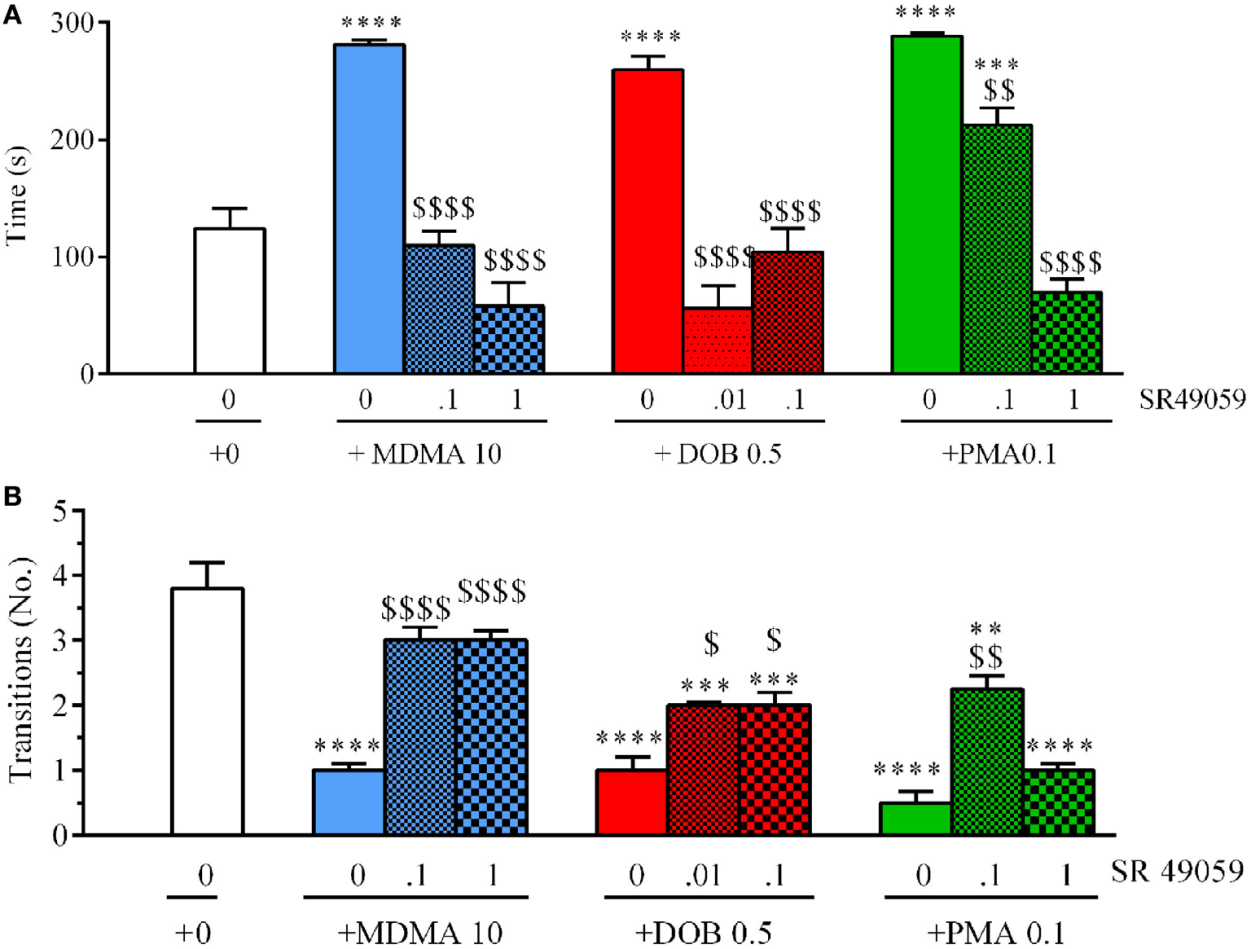
SR49050 dose-dependently reduces the anxiolytic effect induced by 3,4-methylenedioxymethamphetamine (MDMA), DOB, or *para*-methoxyamphetamine (PMA) in the novel tank diving test. The time spent in the upper half of the tank **(A)** and the number of transitions from the lower half to the upper half **(B)** were recorded during the 5-min sessions. The combination of SR49059 (ng/kg) or vehicle and each drug (mg/kg) was given intramuscularly (IM) immediately before the test. Mean values ± SEM; *n* = 10 fish per group. **p < 0.01, ***p < 0.001, ****p < 0.0001 vs. the corresponding saline group (0 + 0);^$^ *p* < 0.05,^$$^ *p* < 0.01, ^$$$$^*p* < 0.0001 vs. the corresponding drug alone (Tukey’s *post hoc* test).

There were significant between-group differences in the time spent in the upper half of the novel tank during the 5 min after treatment (Figure 5A): MDMA (*F*_3, 36_ = 42.99, *p* < 0.0001), DOB (*F*_3, 36_ = 25.45, *p* < 0.0001), and PMA (*F*_3, 36_ = 54.81, *p* < 0.0001). *Post hoc* analysis showed that treatment with the drugs alone significantly increased the time spent in the upper half in comparison with vehicle group, but this behavior was blocked by the coadministration of SR49059. Acute treatment with MDMA, DOB, and PMA decreased the number of transitions from the lower to the upper half of the tank during the 5 min after treatment: MDMA (*F*_3, 36_ = 24.54, *p* < 0.0001), DOB (*F*_3, 36_ = 22.43, *p* < 0.0001), and PMA (*F*_3, 36_ = 35.76, *p* = 0.0001) (Figure 5B). SR49059 antagonized the decrease in the number of transitions induced by the drugs, except for those induced by the highest dose of PMA. SR49059 antagonism was obtained at doses that did not affect the time spent in the upper and lower halves of the tank (*F*_3, 36_ = 0.56, *p* = 0.64) or the number of transitions (*F*_3, 36_ = 0.33, *p* = 0.81) (Table 2).

**Table 2:** Effect of SR40059 on anxiety-like behavior in zebra fish.

Treatment	Dose (ng/kg)	Novel tank diving test (s)	Transitions to upper half (No.)
Vehicle	0	123.80 ± 17.40	3.87 ± 1.40
SR49059	0.01	61.80 ± 23.40	4.00 ± 1.90
SR49059	0.1	102.32 ± 4.86	4.86 ± 1.80
SR49059	1	94.00 ± 37.70	2.25 ± 0.95

**Treatment**	**Dose (ng/kg)**	**Light–dark test (s)**	**Transitions (No.)**

Vehicle	0	−122.70 ± 17.59	58.59 ± 4.19
SR49059	0.01	−74.25 ± 22.31	66.50 ± 16.60
SR49059	0.1	−74.50 ± 4.85	37.5 ± 9.30

*Mean values ± SEM (*n* = 10 for each treatment)*.

### Light–Dark Test

**Figure 6:**
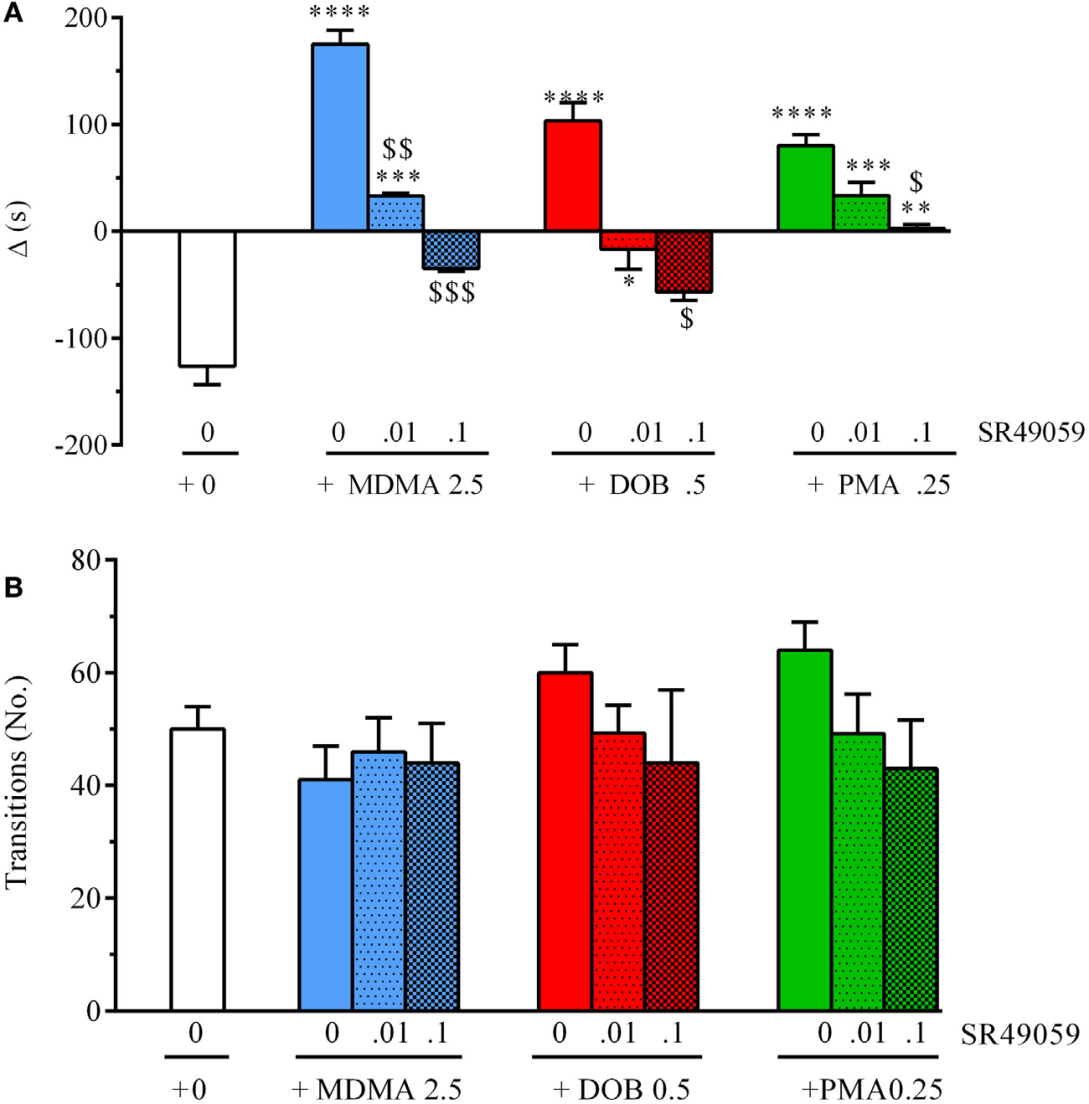
SR49050 dose-dependently blocks the anxiolytic effect induced by 3,4-methylenedioxymethamphetamine (MDMA), 2,5-dimethoxy-4-bromo-amphetamine hydrobromide (DOB), and *para*-methoxyamphetamine (PMA) in the light dark test. Mean values ± SEM of the differences (Δ) in the time spent in the light and dark compartments **(A)** and the number of transitions between them **(B)** during the 5-min sessions. The combination of SR49059 (ng/kg) or vehicle and each drug (mg/kg) was given intramuscularly (IM) immediately before each test. *n* = 10 fish per group. *p < 0.05, **p < 0.01, ***p < 0.001, ****p < 0.0001 vs. the corresponding saline group (0 + 0); ^$^*p* < 0.05, ^$$^*p* < 0.01, ^$$$^ *p* < 0.001 vs. the corresponding drug alone (Tukey’s test).

The difference in the time spent in the white and black compartments after treatment revealed between-group differences in emotional-like behavior: MDMA (*F*_3, 36_ = 19.304, *p* < 0.0001), DOB (*F*_3, 36_ = 13.06, *p* < 0.0001), and PMA (*F*_3, 36_ = 19.37, *p* < 0.0001) (Figure 6A). *Post hoc* analysis showed that treatment with the drugs alone significantly increased the time spent in the white compartment in comparison with the vehicle group, but the addition of SR49059 reduced this time in a dose-dependent manner. The increased time spent in the light–dark compartment was not due to motor impairment as there was no change in the number of transitions from one compartment to the other: MDMA (*F*_3, 36_ = 0.42, *p* = 0.74), DOB (*F*_3, 36_ = 0.77, *p* = 0.52), and PMA (*F*_3, 36_ = 1.9, *p* = 0.14) (Figure 6B). When given alone, SR49049 did not affect either parameter (time: *F*_2, 27_ = 1.83, *p* = 0.18; transitions: *F*_2, 27_ = 2.29, *p* = 0.12) (Table 2).

### Brain IT Levels

**Figure 7:**
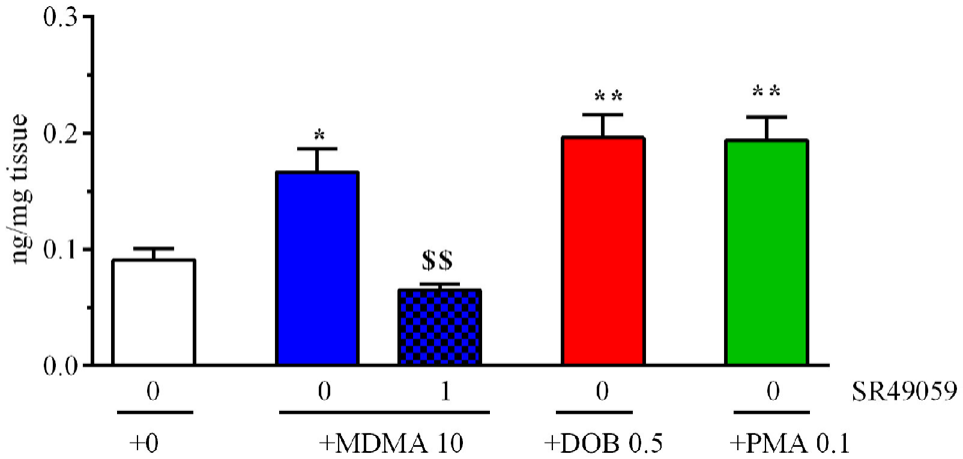
3,4-Methylenedioxymethamphetamine (MDMA), 2,5-dimethoxy-4-bromo-amphetamine hydrobromide (DOB), or *para*-methoxyamphetamine (PMA) significantly increased cerebral IT levels 5 min after treatment. Mean values ± SEM of three to four samples per group. The combination of SR49059 (1 ng/kg) and MDMA (mg/kg) significantly reduced IT levels. *p < 0.05, **p < 0.01 vs. the corresponding saline group (0 + 0); ^$$^*p* < 0.01 vs. MDMA alone (Tukey’s test).

The drugs significantly increased brain IT levels in comparison with the vehicle group (*F*_4, 25_ = 13.88, *p* < 0.0001) (Figure 7), whereas the coadministration of SR49059 and MDMA significantly reduced the MDMA-induced increase.

## Discussion

This study investigated the modulatory role of V_1a_-like subtype receptors on MDMA-, DOB-, and PMA-induced rewarding, prosocial, and anxiolytic effects in zebra fish. The selective antagonist of vasopressin V_1_a subtype receptors, SR49059, reduced the effects induced by all of the tested drugs (which were associated with increased IT concentrations in the brain), whereas SR49059 completely blocked the brain IT release induced by MDMA.

It has been previously shown that SR49059 blocks the prosocial and anxiolytic effects induced by the injection of neurohypophyseal OT/AVP hormones and their teleost fish homologs IT/AVT (60). AVT receptors have been identified in non-mammalian vertebrates such as teleosts, and it has been shown that they are involved in water balance, osmotic homeostasis, sociality, aggression and sexual behavior (68, 69). Although teleost fish receptors have not yet been fully characterized, like mammalian OT and V_1a_/V_1_b receptor subtypes, AVT and IT receptors may act through a phosopholipase C/inositol 1,4,5-trisphosphate intracellular signaling pathway (70). It has been previously shown (71) that SR49059 is a more selective and potent antagonist of V_1_a than V_1b_ receptors, but its affinity for V_1A_ and OT receptors is similar at least in mice (Ki = 0.94 ± 22 and 13.2 ± 19, respectively). However, further studies are needed to investigate its affinity for zebra fish IT/AVT receptors.

Our findings show that IT/AVT receptors are involved in MDMA-, PMA-, DOB-induced reward in zebra fish, as shown by the reduction in CPP when SR49059 was coadministered with the drugs. Previous studies of the interactions between OT-like systems and the rewarding effects of drugs have found that OT receptor density or mRNA expression change differently depending on the dose (72) and the considered brain area (73–81). The OT antagonist atosiban reduces MDMA-induced drug discrimination in rats, and the OT analog carbetocin partially generalizes to the MDMA training cue (82), thus suggesting that OT receptor activation is a major factor in the subjective effects of MDMA. It is known that OT modulates DA turnover, and it has been shown that OT receptors functionally interact with DA D_2_ receptors in the NAc, one of the most important brain areas for reward (83). On the basis of these findings, there is increasing interest in using OT to treat alcohol and nicotine dependence (84, 85). A significant rewarding effect has been observed when a high dose of SR49059 is administered alone. As suggested by Baracz et al. (54) who found that the OT receptor antagonist desGly-NH2,d(CH2)5[D-Tyr2,Thr4] had a similar effect on rats undergoing the CPP test, a tonic level of endogenous neurohyphophyseal hormones could be a contributory factor, but more studies are needed to clarify the mechanism.

Our social preference test findings show that the IT/AVT system also modulates the social behavior of zebra fish as the coadministration of SR49059 with MDMA, DOB, or PMA significantly blocked the increased sociability induced by the drugs alone. Drug abuse is closely associated with social contexts in which OT plays an important role. No data are available concerning PMA and DOB, but the prosocial effects of MDMA have been associated with central OT release in rat and human studies (18). MDMA increases plasma OT levels in human MDMA users at dance parties and in humans given MDMA in placebo-controlled laboratory experiments, and this increase has been related to increased subjective feelings of sociability (57, 86). The increased OT levels have been attributed to the ability of MDMA to release serotonin via hypothalamic 5-HT terminals apposed to OT-containing perikarya (87). The 5-HT_1A_ antagonist WAY 100,635 reduces the increase in plasma OT levels and the adjacent lying behavior of rats induced by MDMA (88). In line with this, the selective OT receptor antagonist L-3668999 abolishes the prosocial effects of MDMA in mice, and quantitative analyses of brain proteome have revealed changes in 21 proteins associated with sociability (89). In addition, the MDMA-induced increase in rodent prosocial behavior is prevented by the same dose of WAY 100,635 and the 5-HT_2B_/_2C_ receptor antagonist SB 206553 (90), thus suggesting that the prosocial effect of MDMA in rodents may be mediated by OT release as a result of 5-HT_1A_ and 5-HT_2B/2C_ receptor interactions. The involvement of 5-HT_2A/2C_ serotonin receptors in the social preferences of zebra fish has recently been confirmed by the blockade obtained using ritanserin coadministered with MDMA, DOB, or PMA (23). Treatment with the vasopressin V_1_a antagonist SR49059 attenuates the increase in adjacent lying elicited in rats by MDMA, OT, and AVP, thus suggesting that a common mechanism mediated by V_1_a receptors underlies their prosocial behavioral effects (56). It has also been shown that the ICV administration of OT reverses the social deficits observed in OT receptor knockout mice and that this is also probably due to the functional activity of OT at V_1_a receptors (91). It is thought that the prosocial effects of OT are at least partially mediated by its ability to alleviate anxiety. We used both the novel tank diving test and the light–dark test to evaluate emotional-like behavior as previous studies have shown that one test alone does not adequately capture the information necessary to interpret the results (92, 93). In both paradigms, our findings indicate that SR49059 reduced the anxiolytic effects induced by MDMA, DOB, and PMA. The reduced diving and bottom dwelling observed in our tank diving experiments after this treatment is in line with the typical top dwelling responses induced by MDMA dissolved in tank water (40 and 80 mg/L) (44). The possibility that the SR49059-induced blockade of the anxiolytic effects of the drugs was due to altered locomotion can be excluded because the number of transitions from one level to the other by the control and treated zebra fish was similar.

The biochemical evaluation of brain IT levels after MDMA, PMA, and DOB treatment revealed a significant increase in comparison with the vehicle group, when using the highest active dose of each drug: one of the limitations of this study is that IT levels were not evaluated after increasing drug doses. However, it was found that the coadministration of SR49059 significantly blocked IT release, thus supporting the involvement of the IT/AVT system in the drug-elicited effects. Further studies are now needed to evaluate IT release after the combined administration of SR49059 and DOB/PMA, and after treatment with increasing doses of each compound. The only previously published study of brain nonapeptide levels in zebra fish (67) evaluated the relationship between social status and the IT/AVT system in different brain areas, and found that nonapeptide levels rapidly increased after an acute social interaction. Our data concerning MDMA are in line with those of Forsling et al. (94), who found that the presence of MDMA is associated with a dose-dependent increase in OT and AVP release in isolated rat hypothalami.

The mechanisms underlying the MDMA-, DOB-, and PMA-induced increase in brain IT levels, and the SR49059-induced blockade of this effect of MDMA, are not clear. SR49059 has more affinity for V_1a_ than V_1b_ or V_2_ receptors, and a weak affinity for OT receptors (71). Our findings suggest that MDMA (and perhaps also DOB and PMA) indirectly stimulates V_1a_ receptors as a result of serotonin-induced OT/IT and/or AVP/AVT release in the hypothalamus, as previously suggested by Jørgensen et al. (58). It has been reported that 5-HT, serotonin precursors, serotonin releasers, serotonin reuptake inhibitors and serotonin receptor agonists stimulate the release of vasopressin and OT into peripheral blood (58). Furthermore, 5-HT stimulates vasopressin secretion via 5-HT_2A_ and 5-HT_2C_ receptors, whereas 5-HT_1A_ receptors are also involved in OT secretion (58). In zebra fish, 5-HT_1A_-like, 5-HT_1B_ and 5-HT_2_ receptors have been found in homologous regions in the brain, including the hypothalamus (95, 96), where it has been shown that neuropeptides and 5-HT receptors are associated with fear and anxiety (97–99). The decrease of IT operated by SR49059 in combination with MDMA appears difficult to be explained with a simple effect through V_1a_ receptors. It can be argued that SR49059 acts directly to inhibit IT release through 5-HT receptors. Experiments using 5HT receptor antagonists on IT release could better explain the mechanism. In line of our findings, SR49059 partially reversed MDMA-induced increases in adjacent lying, a measure of social behavior in rats (56).

In conclusion, our findings show for the first time that there is an interaction between IT/OT and the rewarding, social, and anxiolytic effects of PMA and DOB in zebra fish. Taken together, they suggest that the IT/OT system may be an important target for the development of new pharmacotherapies for the treatment of the misuse of MDMA and its phenetylamine derivatives and related affective disorders (sociability, anxiety). Although further studies are required to clarify the mechanism underlying the effects of the OT system on drug abuse, the findings of this study support the view that that the zebra fish model is a highly sensitive means of screening new OT-like agonists and antagonists.

## Ethics Statement

Experimental procedures were carried out in accordance with the European Community Council Directive No. 86/609/EEC and the subsequent Italian Law on the Protection of animals used for experimental and other scientific reasons. The experimental protocol was approved by the Italian Governmental Decree No. 18/2013 and 34/2017.

## Author Contributions

All of the authors meet all of the criteria for authorship and significantly contributed to the research described in this article. MS and DB developed and planned the experiment. LP performed the behavioral experiments. GB performed the HPLC experiments. MS and DB were responsible for all of the statistical analyses. MS and DB wrote the manuscript.

## Conflict of Interest Statement

All of the authors declare that they do not have any commercial or financial relationships that could be interpreted as reflecting a conflict of interest. The handling editor declares a shared affiliation, though no collaboration with one of the authors, and states that the process met the standards of a fair and objective review.

## Abbreviations

5 HT_2A/C_: serotonin receptor type 5, subtype A and C; AVP, arginine-vasopressin system; AVT: arginine vasotocin; DOB: 2,5-dimethoxy-4-bromo-amphetamine hydrobromide; IM: intramuscularly; IT: isotocin; MDMA: 3,4-methylenedioxymethamphetamine; OT: oxytocin; NAc: nucleus accumbens; PMA, *para*-methoxyamphetamine; SR49059: ((2S)-1-[[(2R,3S) 5-chloro-3(2-chlorophenyl)-1-[(3,4-dimethoxyphenyl) sulfonyl]-2,3-dihydro-3-hydroxy-1H-indol-2-l]carbonyl]-2-pyrrolidine carboxamide); V_1a_: vasopressin receptor, type1a; V_1_b: vasopressin receptor, type1b.

## References

[1] McCreary AC Müller CP Filip M Psychostimulants: basic and clinical pharmacology. Int Rev Neurobiol (2015) 120:41–83. 10.1016/bs.irn.2015.02.008 26070753

[2] Hayase T Yamamoto Y Yamamoto K. Persistent anxiogenic effects of a single or repeated doses of cocaine and methamphetamine: interactions with endogenous cannabinoid receptor ligands. Behav Pharmacol (2005) 16:395–404. 10.1186/1471-2210-11-6 16148444

[3] Hayase T Yamamoto Y Yamamoto K. Behavioral effects of ketamine and toxic interactions with psychostimulants. BMC Neurosci (2006) 7:25. 10.1186/1471-2202-7-25 16542420 PMC1473192

[4] McGregor IS Gurtman CG Morley KC Clemens KJ Blokland A Li KM Increased anxiety and depressive symptoms months after MDMA (ecstasy) in rats: drug-induced hyperthermia does not predict long-term outcomes. Psychopharmacology (Berl) (2003) 168:465–74. 10.1007/s00213-003-1452-8 12700882

[5] Navarro JF Rivera A Maldonado E Cavas M de la Calle A Anxiogenic-like activity of 3,4-methylenedioxy-methamphetamine (ecstasy) in the social interaction test is accompanied by an increase of c-fos expression in mice amygdala. Prog Neuropsychopharmacol Biol Psychiatry (2004) 28:249–54. 10.1016/j.pnpbp.2003.10.016 14751419

[6] Schutová B Hrubá L Pometlová M Deykun K Slamberová R. Cognitive functions and drug sensitivity in adult male rats prenatally exposed to methamphetamine. Physiol Rev (2009) 58:741–50. 19093723 10.33549/physiolres.931562

[7] Williams MT Moran MS Vorhees CV. Refining the critical period for methamphetamine-induced spatial deficits in the Morris water maze. Psychopharmacology (Berl) (2003) 168:329–38. 10.1007/s00213-003-1433-y 12684734

[8] Thompson VB Heiman J Chambers JB Benoit SC Buesing WR Norman MK Long-term behavioral consequences of prenatal MDMA exposure. Physiol Behav (2008) 96:593–601. 10.1016/j.physbeh.2008.12.013 19162054 PMC2649789

[9] Montoya AG Sorrentino R Lukas SE Price BH Long-term neuropsychiatric consequences of “ecstasy” (MDMA): a review. Rev Psychiatry (2002) 10:212–20. 12119307

[10] European Monitoring Centre for Drugs and Drug Addiction. Report on the Risk of PMMA in the Framework of the Joint Action on New Synthetic Drugs (2003). Available from: http://www.emcdda.europa.eu/publications/risk-assessments/pmma

[11] Carroll FI Lewin AH Mascarella SW Seltzman HH Reddy PA. Designer drugs: a medicinal chemistry perspective. Ann N Y Acad Sci (2012) 1248:18–38. 10.1111/j.1749-6632.2011.06199.x 22092008

[12] United Nations Office on Drugs and Crime. World Drug Report. Vienna: United Nations Publication (2015). Available from: https://www.unodc.org/documents/wdr2015/World_Drug_Report_2015.pdf

[13] Hill SL Thomas SH. Clinical toxicology of newer recreational drugs. Clin Toxicol (Phila) (2011) 49:705–19. 10.3109/15563650.2011.615318 21970769

[14] Lawn W Barratt M Williams M Horne A Winstock A The NBOMe hallucinogenic drug series: patterns of use, characteristics of users and self-reported effects in a large international sample. J Psychopharmacol (2014) 28:780–8. 10.1177/0269881114523866 24569095

[15] Chen A The Underground Website Where You Can Buy Any Drug Imaginable (2011). Available from: www.pearltrees.com/u/69792968-underground-website-imaginable 8160

[16] Gonçalves J Baptista S Silva AP. Psychostimulants and brain dysfunction: a review of the relevant neurotoxic effects. Neuropharmacology (2014) 87:135e149. 10.1016/j.neuropharm.2014.01.006 24440369

[17] Callaghan PD Owens WA Javors MA Sanchez TA Jones DJ Irvine RJ In vivo analysis of serotonin clearance in rat hippocampus reveals that repeated administration of p-methoxyamphetamine (PMA), but not 3,4-methylenedioxymethamphetamine (MDMA), leads to long-lasting deficits in serotonin transporter function. J Neurochem (2007) 100:617e627. 10.1111/j.1471-4159.2006.04247.x 17181558

[18] Kamilar-Britt P Bedi G The prosocial effects of 3,4-methylenedioxy methamphetamine (MDMA): controlled studies in humans and laboratory animals. Neurosci Biobehav Rev (2015) 57:433–46. 10.1016/j.neubiorev.2015.08.016 26408071 PMC4678620

[19] Sumnall HR O’Shea E Marsden CA Cole JC. The effects of MDMA pretreatment on the behavioural effects of other drugs of abuse in the rat elevated plus-maze test. Pharmacol Biochem Behav (2004) 77:805–14. 10.1016/j.pbb.2004.02.007 15099927

[20] Faria R Magalhães A Monteiro PR Gomes-Da-Silva J Amélia Tavares M Summavielle T. MDMA in adolescent male rats: decreased serotonin in the amygdala and behavioral effects in the elevated plus-maze test. Ann N Y Acad Sci (2006) 1074:643–9. 10.1196/annals.1369.062 17105959

[21] Ho YJ Pawlak CR Guo L Schwarting RK. Acute and long-term consequences of single MDMA administration in relation to individual anxiety levels in the rat. Behav Brain Res (2004) 149:135–44. 10.1016/S0166-4328(03)00220-1 15129777

[22] Lin HQ Burden PM Christie MJ Johnston GA. The anxiogenic-like and anxiolytic-like effects of MDMA on mice in the elevated plus-maze: a comparison with amphetamine. Pharmacol Biochem Behav (1999) 62:403–8. 10.1016/S0091-3057(98)00191 10080230

[23] Ponzoni L Sala M Braida D. Ritanserin-sensitive receptors modulate the prosocial and the anxiolytic effect of MDMA derivatives, DOB and PMA, in zebrafish. Behav Brain Res (2016) 314:181–9. 10.1016/j.bbr.2016.08.009 27506653

[24] Greer G Tolbert R Subjective reports of the effects of MDMA in a clinical setting. J Psychoactive Drugs (1986) 18:319–27. 10.1080/02791072.1986.10472364 2880946

[25] Schifano F Chronic atypical psychosis associated with MDMA (“ecstasy”) abuse. Lancet (1991) 338:1335 10.1016/0140-6736(91)92633-D 1682711

[26] Mithoefer MC Grob CS Brewerton TD. Novel psychopharmacological therapies for psychiatric disorders: psilocybin and MDMA. Lancet Psychiatry (2016) 3:481–8. 10.1016/S2215-0366(15)00576-3 27067625

[27] Shulgin A Shulgin A PIHKAL, A Chemical Love Story. Berkeley, CA: Transform Press (1998). 978 p.

[28] Daws LC Irvine RJ Callaghan PD Toop NP White JM Bochner F Differential behavioural and neurochemical effects of paramethoxy-amphetamine and 3,4-methylenedioxymethamphetamine in the rat. Prog Neuropsychopharmacol Biol Psychiatry (2000) 24:955–77. 10.1016/S0278-5846(00)00113-5 11041537

[29] Acuna-Castillo C Villalobos C Moya PR Saez P Cassels BK Huidobro-Toro JP Differences in potency and efficacy of a series of phenylisopropylamine/phenylethylamine pairs at 5-HT2A and 5-HT2C receptors. Br J Pharmacol (2002) 136:510–9. 10.1038/sj.bjp.0704747 12055129 PMC1573376

[30] Glennon RA Titeler M Lyon RA. A preliminary investigation of the psychoactive agent 4-bromo-2,5-dimethoxyphenethylamine: a potential drug of abuse. Pharmacol Biochem Behav (1988) 30:597–601. 10.1016/0091-3057(88)90071-8 3211969

[31] Monte AP Marona-Lewicka D Parker MA Wainscott DB Nelson DL Nichols DE Dibenzofuran analogues of hallucinogens. Three models of 4-substituted (2,5-dimethoxyphenyl) alkylamine derivatives with rigidified methoxy groups. J Med Chem (1996) 39:2953–61. 10.1021/jm960199j 8709129

[32] Balíková M Nonfatal and fatal DOB (2,5-dimethoxy-4-bromampheta-mine) overdose. Forensic Sci Int (2005) 153:85–91. 10.1016/j.forsciint.2005.04.02 15979834

[33] Spinella M Psychopharmacology of Herbal Medicine. Cambridge: The MIT Press (2001). 590 p.

[34] Delliou D. 4-Bromo-2,5-dimethoxyamphetamine: psychoactivity, toxic effects and analytical methods. Forensic Sci Int (1983) 21:259–67. 10.1016/0379-0738(83)90131-7 6873782

[35] Buhrich N Morris G Cook G. Bromo-DMA: the Australasian hallucinogen? Aust N Z J Psychiatry (1983) 17:275–9. 10.3109/00048678309161284 6580896

[36] Winek CL Collom WD Bricker JD A death due to 4-bromo-2,5-dimethoxyamphetamine. Clin Toxicol (1981) 18:267–71. 10.3109/15563658108990035 7237958

[37] Martin TL. Three cases of fatal paramethoxyamphetamine overdose. J Anal Toxicol (2001) 25:649–51. 10.1093/jat/25.7.649 11599618

[38] Ling LH Marchant C Buckley NA Prior M Irvine RJ Poisoning with the recreational drug paramethoxyamphetamine (“death”). Med J Aust (2001) 174:453–5. 11386590 10.5694/j.1326-5377.2001.tb143372.x

[39] Johansen SS Hansen AC Müller IB Lundemose JB Franzmann MB. Three fatal cases of PMA and PMMA poisoning in Denmark. J Anal Toxicol (2003) 27:253–6. 10.1093/jat/27.4.253 12820749

[40] Caldicott DG Edwards NA Kruys A Kirkbride KP Sims DN Byard RW Dancing with “death”: p-methoxyamphetamine overdose and its acute management. J Toxicol Clin Toxicol (2003) 41:143–54. 10.1081/CLT-120019130 12733852

[41] Gołembiowska K Jurczak A Kamińska K Noworyta-Sokołowska K Górska A. Effect of some psychoactive drugs used as ‘legal highs’ on brain neurotransmitters. Neurotox Res (2016) 29:394–407. 10.1007/s12640-015-9569-1 26501352 PMC4786600

[42] Kyzar EJ Kalueff AV. Exploring hallucinogen pharmacology and psychedelic medicine with zebrafish models. Zebrafish (2016) 13:379–90. 10.1089/zeb.2016.1251 27002655

[43] Neelkantan N Mikhaylova A Stewart AM Arnold R Gjeloshi V Kondaveeti D Perspectives on zebrafish models of hallucinogenic drugs and related psychotropic compounds. ACS Chem Neurosci (2013) 4:1137–50. 10.1016/S0379-0738(00)00425-4 23883191 PMC3750682

[44] Stewart A Riehl R Wong K Green J Cosgrove J Vollmer K Behavioral effects of MDMA (’ecstasy’) on adult zebrafish. Behav Pharmacol (2011) 22:275–80. 10.1097/FBP.0b013e328345f758 21522057 PMC3083639

[45] Braida D Limonta V Pegorini S Zani A Guerini-Rocco C Gori E Hallucinatory and rewarding effect of salvinorin A in zebrafish: kappa-opioid and CB1-cannabinoid receptor involvement. Psychopharmacology (Berlin) (2007) 190:441–8. 10.1007/s00213-006-0639-1 17219220

[46] Levin ED Bencan Z Cerutti DT Anxiolytic effects of nicotine. Physiol Behav (2007) 90:54–8. 10.1016/j.physbeh.2006.08.026 17049956

[47] Ng MC Yang YL Lu KT. Behavioral and synaptic circuit features in a zebrafish model of fragile X syndrome. PLoS One (2013) 8:e51456. 10.1371/journal.pone.0051456 23536755 PMC3594205

[48] Collins CJ The Zebrafish (Danio rerio) as a Highly Sensitive Tool for Screening Hallucinogenic Drug Action: Lessons from Mescaline and Salvinorin A [Dissertation]. New Orleans, LA: Neuroscience Program, Tulane University (2012).

[49] Cachat J Kyzar EJ Collins C Gaikwad S Green J Roth A Unique and potent effects of acute ibogaine on zebrafish: the developing utility of novel aquatic models for hallucinogenic drug research. Behav Brain Res (2013) 236:258–69. 10.1016/j.bbr.2012.08.041 22974549

[50] Kyzar EJ Collins C Gaikwad S Green J Roth A Monnig L Effects of hallucinogenic agents mescaline and phencyclidine on zebrafish behavior and physiology. Prog Neuropsychopharmacol Biol Psychiatry (2012) 37:194–202. 10.1016/j.pnpbp.2012.01.00.3 22251567 PMC3294104

[51] Zakhary SM Ayubcha D Ansari F Kamran K Karim M Leheste JR A behavioral and molecular analysis of ketamine in zebrafish. Synapse (2011) 65:160–7. 10.1002/syn.20830 20623473 PMC2978795

[52] Grossman L Utterback E Stewart A Gaikwad S Chung KM Suciu C Characterization of behavioral and endocrine effects of LSD on zebrafish. Behav Brain Res (2010) 214:277–84. 10.1016/j.bbr.2010.05.039 20561961

[53] Ponzoni L Braida D Sala M Abuse potential of methylenedioxymethamphetamine (MDMA) and its derivatives in zebrafish: role of serotonin 5HT2-type receptors. Psychopharmacology (Berl) (2016) 233:3031–9. 10.1007/s00213-016-4352-4 27318987

[54] Baracz SJ Cornish JL. The neurocircuitry involved in oxytocin modulation of methamphetamine addiction. Front Neuroendocrinol (2016) 43:1–18. 10.1016/j.yfrne.2016.08.001 27546878

[55] Qi J Yang JY Wang F Zhao YN Song M Wu CF Effects of oxytocin on methamphetamine-induced conditioned place preference and the possible role of glutamatergic neurotransmission in the medial prefrontal cortex of mice in reinstatement. Neuropsychopharmacology (2013) 38:2249–59. 10.1038/npp.2013 19371575

[56] Ramos L Hicks C Kevin R Caminer A Narlawar R Kassiou M Acute prosocial effects of oxytocin and vasopressin when given alone or in combination with 3,4-methylenedioxymethamphetamine in rats: involvement of the V_1A_ receptor. Neuropharmacology (2013) 38:2249–59. 10.1038/npp.2013.125 23676791 PMC3773675

[57] Dumont GJ Sweep FC van der Steen R Hermsen R Donders AR Touw DJ Increased oxytocin concentrations and prosocial feelings in humans after ecstasy (3,4-methylenedioxymethamphetamine) administration. Soc Neurosci (2009) 4:359–66. 10.1080/17470910802649470 19562632

[58] Jørgensen H Riis M Knigge U Kjaer A Warberg J. Serotonin receptors involved in vasopressin and oxytocin secretion. J Neuroendocrinol (2003) 15:242–9. 10.1046/j.1365-2826.2003.00978.x 12588512

[59] Goodson JL Bass AH Social behavior functions and related anatomical characteristics of vasotocin/vasopressin systems in vertebrates. Brain Res Brain Res Rev (2001) 35:246–65. 10.1016/S0165-0173(01)00043-1 11423156

[60] Braida D Donzelli A Martucci R Capurro V Busnelli M Chini B Neurohypophyseal hormones manipulation modulate social and anxiety-related behavior in zebrafish. Psychopharmacology (2012) 220:319–23. 10.1007/s00213-011-2482-2 21956239

[61] Braida D Donzelli A Martucci R Ponzoni L Pauletti A Sala M. Neurohypophyseal hormones protect against pentylenetetrazole-induced seizures in zebrafish: role of oxytocin-like and V1a-like receptor. Peptides (2012) 37:327–33. 10.1016/j.peptides.2012.07.013 22828174

[62] Chini B Manning M Guillon G Affinity and efficacy of selective agonists and antagonists for vasopressin and oxytocin receptors: an “easy guide” to receptor pharmacology. Prog Brain Res (2008) 170:513–7. 10.1016/S0079-6123(08)00438-X 18655904

[63] Cheung E Chatterjee D Gerlai R. Subcutaneous dye injection for marking and identification of individual adult zebrafish (*Danio rerio*) in behavioral studies. Behav Res Methods (2014) 46:619–24. 10.3758/s13428-013-0399-x 24057277

[64] Saverino C Gerlai R. The social zebrafish: behavioral responses to conspecific, heterospecific, and computer animated fish. Behav Brain Res (2008) 191:77–87. 10.1016/j.bbr.2008.03.013 18423643 PMC2486438

[65] Engeszer RE Ryan MJ Parichy DM. Learned social preference in zebrafish. Curr Biol (2004) 14:881–4. 10.1016/j.cub.2004.04.042 15186744

[66] Egan RJ Bergner CL Hart PC Cachat JM Canavello PR Elegante MF Understanding behavioral and physiological phenotypes of stress and anxiety in zebrafish. Behav Brain Res (2009) 205:38–44. 10.1016/j.bbr.2009.06.022 19540270 PMC2922906

[67] Teles MC Gozdowska M Kalamarz-Kubiak H Kulczykowska E Oliveira RF. Agonistic interactions elicit rapid changes in brain nonapeptide levels in zebrafish. Horm Behav (2016) 84:57–63. 10.1016/j.yhbeh.2016.05.020 27235811

[68] Filby AL Paull GC Hickmore TF Tyler CR. Unravelling the neurophysiological basis of aggression in a fish model. BMC Genomics (2010) 11:498. 10.1186/1471-2164-11-498 20846403 PMC2996994

[69] Lema SC. Identification of multiple vasotocin receptor cDNAs in teleost fish: sequences, phylogenetic analysis, sites of expression, and regulation in the hypothalamus and gill in response to hyperosmotic challenge. Mol Cell Endocrinol (2010) 321(2):215–30. 10.1016/j.mce.2010.02.015 20167249

[70] Warne JM. Cloning and characterization of an arginine vasotocin receptor from the euryhaline flounder *Platichthys flesus*. Gen Comp Endocrinol (2001) 122:312–9. 10.1006/gcen.2001.7644 11356043

[71] Busnelli M Bulgheroni E Manning M Kleinau G Chini B. Selective and potent agonists and antagonists for investigating the role of mouse oxytocin receptors. J Pharmacol Exp Ther (2013) 346:318–27. 10.1124/jpet.113.202994 23723434 PMC3716315

[72] Zanos P Wright SR Georgiou P Yoo JH Ledent C Hourani SM Chronic methamphetamine treatment induces oxytocin receptor up-regulation in the amygdala and hypothalamus via an adenosine A2a receptor-independent mechanism. Pharmacol Biochem Behav (2014) 119:72–9. 10.1016/j.pbb.2013.05.009 23680573

[73] Zanos P Georgiou P Weber C Robinson F Kouimtsidis C Niforooshan R Oxytocin and opioid addiction revisited: old drug, new applications. Br J Pharmacol (2017). 10.1111/bph.13757 28378414 PMC6016632

[74] Georgiou P Zanos P Garcia-Carmona JA Hourani S Kitchen I Laorden ML Methamphetamine abstinence induces changes in µ-opioid receptor, oxytocin and CRF systems: association with an anxiogenic phenotype. Neuropharmacology (2016) 105:520–32. 10.1016/j.neuropharm.2016.02.012 26896754

[75] Sarnyai Z Vecsernyés M Laczi F Bíró E Szabó G Kovács GL. Effects of cocaine on the contents of neurohypophyseal hormones in the plasma and in different brain structures in rats. Neuropeptides (1992) 23:27–31. 10.1016/0143-4179(92)90006-I 1407414

[76] Kovàcs GL Sarnyai Z Barbarczi E Szabó G Telegdy G. The role of oxytocin-dopamine interactions in cocaine-induced locomotor hyperactivity. Neuropharmacology (1990) 29:365–8. 10.1016/0028-3908(90)90095-9 2160623

[77] You ZD Li JH Song CY Wang CH Lu CL. Chronic morphine treatment inhibits oxytocin synthesis in rats. Neuroreport (2000) 11:3113–6. 10.1097/00001756-200009280-00015 11043533

[78] Butovsky E Juknat A Elbaz J Shabat-Simon M Eilam R Zangen A Chronic exposure to Delta9-tetrahydrocannabinol downregulates oxytocin and oxytocin-associated neurophysin in specific brain areas. Mol Cell Neurosci (2006) 31:795–804. 10.1016/j.mcn.2006.01.008 16513365

[79] Moaddab M Hyland BI Brown CH. Oxytocin enhances the expression of morphine-induced conditioned place preference in rats. Psychoneuroendocrinology (2015) 53:159–69. 10.1016/j.psyneuen.2015.01.003 25618594

[80] van Ree JM de Wied D Effect of neurohypophyseal hormones on morphine dependence. Psychoneuroendocrinology (1977) 2(1):35–41. 10.1016/0306-4530(77)90029-4 561983

[81] Ibragimov R Kovács GL Szabó G Telegdy G. Microinjection of oxytocin into limbic-mesolimbic brain structures disrupts heroin self-administration behavior: a receptor-mediated event? Life Sci (1987) 41:1265–71. 10.1016/0024-3205(87)90205-0 3041139

[82] Broadbear JH Tunstall B Beringer K. Examining the role of oxytocin in the interoceptive effects of 3,4-methylenedioxymethamphetamine (MDMA, ‘ecstasy’) using a drug discrimination paradigm in the rat. Addict Biol (2011) 16:202–14. 10.1111/j.1369-1600.2010.00267.x 21070509

[83] Romero-Fernandez W Borroto-Escuela DO Agnati LF Fuxe K Evidence for the existence of dopamine D2-oxytocin receptor heteromers in the ventral and dorsal striatum with facilitatory receptor-receptor interactions. Mol Psychiatry (2013) 18:849–50. 10.1038/mp.2012.103 22824810

[84] Lee MR Weerts EM. Oxytocin for the treatment of drug and alcohol use disorders. Behav Pharmacol (2016) 27:640–8. 10.1097/FBP.0000000000000258 27603752 PMC5089904

[85] Lee H Jang M Noh J Oxytocin attenuates aversive response to nicotine and anxiety-like behavior in adolescent rats. Neurosci Res (2016) 115:29–36. 10.1016/j.neures.2016.11.007 27866932

[86] Wolff K Tsapakis EM Winstock AR Hartley D Holt D Forsling ML Vasopressin and oxytocin secretion in response to the consumption of ecstasy in a clubbing population. J Psychopharmacol (2006) 20:400–10. 10.1177/0269881106061514 16574714

[87] Emiliano AB Cruz T Pannoni V Fudge JL. The interface of oxytocin-labeled cells and serotonin transporter-containing fibers in the primate hypothalamus: a substrate for SSRIs therapeutic effects? Neuropsychopharmacology (2007) 32:977–88. 10.1038/sj.npp.1301206 17035935

[88] Thompson MR Callaghan PD Hunt GE Cornish JL McGregor IS A role for oxytocin and 5-HT(1A) receptors in the prosocial effects of 3,4-methylenedioxy methamphetamine (“ecstasy”). Neuroscience (2007) 146:509–14. 10.1016/j.neuroscience.2007.02.032 17383105

[89] Kuteykin-Teplyakov K Maldonado R. Looking for prosocial genes: ITRAQ analysis of proteins involved in MDMA-induced sociability in mice. Eur Neuropsychopharmacol (2014) 24:1773–83. 10.1016/j.euroneuro.2014.08.007 25241352

[90] Morley KC Arnold JC McGregor IS. Serotonin (1A) receptor involvement in acute 3,4-methylenedioxymethamphetamine (MDMA) facilitation of social interaction in the rat. Prog Neuropsychopharmacol Biol Psychiatry (2005) 29:648–57. 10.1016/j.pnpbp.2005.04.009 15908091

[91] Sala M Braida D Lentini D Busnelli M Bulgheroni E Capurro V Pharmacologic rescue of impaired cognitive flexibility, social deficits, increased aggression, and seizure susceptibility in oxytocin receptor null mice: a neurobehavioral model of autism. Biol Psychiatry (2011) 69:875–82. 10.1016/j.biopsych.2010.12.022 21306704

[92] Blaser RE Rosemberg DB Measures of anxiety in zebrafish (*Danio rerio*): dissociation of black–white preference and novel tank test. PLoS One (2012) 7(5):e36931 10.1371/journal.pone.0036931 22615849 PMC3355173

[93] Maximino C Benzecry R Matos Oliveira KR De Jesus Oliveira Batista E Herculano AM Broock Rosemberg D A comparison of the light/dark and novel tank tests in zebrafish. Behaviour (2012) 149:1099–123. 10.1163/1568539X-00003029

[94] Forsling ML Fallon JK Shah D Tilbrook GS Cowan DA Kicman AT The effect of 3,4-methylenedioxymethamphetamine (MDMA, ‘ecstasy’) and its metabolites on neurohypophysial hormone release from the isolated rat hypothalamus. Br J Pharmacol (2002) 135:649–56. 10.1038/sj.bjp.0704502 11834612 PMC1573171

[95] Lillesaar C. The serotonergic system in fish. J Chem Neuroanat (2011) 41:294–308. 10.1016/j.jchemneu.2011.05.009 21635948

[96] Norton WH Folchert A Bally-Cuif L. Comparative analysis of serotonin receptor (HTR1A/HTR1B families) and transporter (slc6a4a/b) gene expression in the zebrafish brain. J Comp Neurol (2008) 511(4):521–42. 10.1002/cne.21831 18839395

[97] Davis M The role of the amygdala in fear and anxiety. Annu Rev Neurosci (1992) 15:353–75. 10.1146/annurev.ne.15.030192.002033 1575447

[98] Graeff FG Guimarães FS De Andrade TG Deakin JF Role of 5-HT in stress, anxiety, and depression. Pharmacol Biochem Behav (1996) 54:129–41. 10.1016/0091-3057(95)02135-3 8728550

[99] Tanaka M Csabafi K Telegdy G. Neurotransmissions of antidepressant-like effects of kisspeptin-13. Regul Pept (2013) 180:1–4. 10.1016/j.regpep.2012.08.017 22999921

